# The Crosstalk Between Signaling Pathways and Cancer Metabolism in Colorectal Cancer

**DOI:** 10.3389/fphar.2021.768861

**Published:** 2021-11-23

**Authors:** Kha Wai Hon, Syafiq Asnawi Zainal Abidin, Iekhsan Othman, Rakesh Naidu

**Affiliations:** Jeffrey Cheah School of Medicine and Health Sciences, Monash University Malaysia, Bandar Sunway, Malaysia

**Keywords:** colorectal cancer, metabolism, metabolic reprogramming, protein kinase, signaling pathways

## Abstract

Colorectal cancer (CRC) is one of the most frequently diagnosed cancers worldwide. Metabolic reprogramming represents an important cancer hallmark in CRC. Reprogramming core metabolic pathways in cancer cells, such as glycolysis, glutaminolysis, oxidative phosphorylation, and lipid metabolism, is essential to increase energy production and biosynthesis of precursors required to support tumor initiation and progression. Accumulating evidence demonstrates that activation of oncogenes and loss of tumor suppressor genes regulate metabolic reprogramming through the downstream signaling pathways. Protein kinases, such as AKT and c-MYC, are the integral components that facilitate the crosstalk between signaling pathways and metabolic pathways in CRC. This review provides an insight into the crosstalk between signaling pathways and metabolic reprogramming in CRC. Targeting CRC metabolism could open a new avenue for developing CRC therapy by discovering metabolic inhibitors and repurposing protein kinase inhibitors/monoclonal antibodies.

## 1 Introduction

Despite the advancement in early detection and medical treatments in recent decades, cancer incidence and mortality continue to increase gradually, as estimated in Global Cancer Statistic (GLOBOCAN) database under The International Agency for Research of Cancer (IARC) and the World Health Organization (WHO). Based on the latest GLOBOCAN 2021 statistics, colorectal cancer (CRC) remains the third most common cancer and the second leading cause of cancer mortality globally (Sung et al., 2021). CRC accounts for 10 percent of 19.3 million new cancer cases and 9.4 percent of about 10 million cancer deaths worldwide (Sung et al., 2021). The high incidence rate of CRC is correlated with genetics, environmental factors, and lifestyle development (Dunlop et al., 2013; Johnson et al., 2013; Rawla et al., 2019). Previous studies estimated that about 65–70 percent of CRC is thoroughly sporadic with no known family history (Samadder et al., 2015). About 25 percent of CRC cases are known to have a family history related to inflammatory bowel syndrome (Yurgelun et al., 2017). Only 5 percent of CRC cases are hereditary CRC syndrome, namely FAP (familial adenomatous polyposis) and Lynch syndrome (Samadder et al., 2015). CRC arises from the glandular epithelial cells of the large intestine when specific cells undergo a series of genetic and epigenetic mutations to become hyper-proliferative and cancerous over time (Ewing et al., 2014; Dekker et al., 2019). These cancerous cells form a benign adenoma at an early stage, in which some progress into carcinoma and metastasize to other organs at the later stage of malignancy (Dekker et al., 2019).

Like any other cancer, CRC cells undergo rewiring of cellular metabolism via the dysregulation of oncogenes and tumor suppressors during carcinogenesis (Brown et al., 2018). Metabolic reprogramming is an essential cancer hallmark in many cancers, including CRC (Cantor and Sabatini, 2012). By altering core metabolism pathways, such as glutaminolysis, glycolysis, lipid synthesis, and mitochondrial oxidation, metabolic reprogramming allows CRC cells to sustain rapid cell proliferation with high demand for energy and biosynthetic precursors to drive tumor development and cancer metastasis (Brown et al., 2018). Regulation of metabolic reprogramming involves a complex network of different signaling pathways in cancer cells, which various protein kinases can regulate (Garcia-Ortega et al., 2017; Lu and Hunter, 2018). Protein kinases (PTKs) are enzymes that selectively modify the biological activity of biomolecules (lipids, proteins, and carbohydrates) via phosphorylation with ATP as the primary source of phosphate (Cheng et al., 2011). Dysregulation of protein kinases has been reported in multiple cancers, such as breast (Templeton et al., 2014), stomach (Shiroki et al., 2017), pancreas (James et al., 2020), and CRC (Asante et al., 2019), to affect metabolic reprogramming of cancer cells through the manipulation of signaling pathways. This review will discuss the importance of protein kinases as crucial regulators of signaling pathways in the metabolic reprogramming of CRC cells. A deeper understanding of the protein kinases and signaling pathways involved in the metabolic reprogramming in CRC will provide insight into discovering new therapeutic targets.

### 2 The Warburg Effect in Colorectal Cancer

In 1924, Otto Warburg discovered that, even in the presence of oxygen, cancer cells preferentially alter the ATP production towards aerobic glycolysis followed by lactic acid fermentation rather than oxidative phosphorylation (OXPHOS) (Devic, 2016). Compared to normal cells, most pyruvate in cancer cells is distributed into lactate fermentation in the cytosol instead of entering the mitochondrial tricarboxylic acid (TCA) cycle and OXPHOS (Vaupel et al., 2019). The unique metabolic phenotype of cancer cells is known as the Warburg effect (Vaupel et al., 2019). In comparison, OXPHOS produces more ATP per molecule of glucose, but aerobic glycolysis produces ATP more rapidly, favoring the actively proliferating cancer cells (Vaupel et al., 2019). Aerobic glycolysis also generates more glycolytic intermediates and pyruvate to synthesize macromolecules and support ATP production (Blanco and Blanco, 2017b). Subsequently, pyruvate is converted into lactate to enter the Krebs cycle and rapidly produce ATP (Blanco and Blanco, 2017b). Other glycolytic intermediates such as glucose-6-phosphate, fructose-6-phosphate, and glyceraldehyde-3-phosphate are converted into macromolecules (nucleotides, phospholipids, and fatty acids) to sustain cell growth and maintenance (Blanco and Blanco, 2017c).

Certain protein kinases are involved in glucose uptake and aerobic glycolysis as rate-limiting enzymes, such as hexokinase (HK), phosphofructokinase (PFK), and pyruvate kinase (PK) (Blanco and Blanco, 2017b). Accumulated evidence shows that dysregulation of these glycolytic regulatory enzymes is vital to modulate the Warburg effect in CRC via different signaling pathways (Brown et al., 2018). Hexokinase (HK) serves as the rate-limiting enzyme to catalyze the first irreversible step of glucose uptake by converting glucose to glucose-6-phosphate (G6P) upon phosphorylation with ATP (Blanco and Blanco, 2017b). Interestingly, G6P then suppresses the activity of HKII through a feedback inhibition mechanism (Blanco and Blanco, 2017b). G6P is the precursor for major pathways of glucose metabolism, including glycolysis, pentose phosphate pathway, oxidative phosphorylation (OXPHOS), and hexosamine biosynthesis pathways (Blanco and Blanco, 2017b). Therefore, HK is regarded as an essential regulator of glucose metabolism. The mammalian HK family has five isoforms: HKI, HKII, HKIII, HKIV, and HKDC1 (HexoKinase Domain-Containing protein 1) (Wilson, 2003; Irwin and Tan, 2008). Among all, HKII is frequently upregulated in multiple cancers, including breast (Sato-Tadano et al., 2013), glioblastoma (Wolf et al., 2011), prostate (Lee et al., 2019), and CRC (Ho and Coomber, 2016). Previous studies report that HKII plays dual cancer-promoting effects by inducing glycolysis and inhibit apoptosis. HKII binds to voltage-dependent anion channel (VDAC) in the outer membrane of mitochondria to suppress apoptosis by closing the permeability transition pores and preventing cytochrome c release (Yuan et al., 2008; Tait and Green, 2013). Notably, growth factor-induced oncogenic signaling pathways, such as EGFR and PI3K/Akt/mTOR signaling, regulate the mitochondrial translocation of HKII (Makinoshima et al., 2014; Liu et al., 2015; Ciscato et al., 2020). Mitochondrial binding of HKII also reduces the feedback inhibition from G6P to increase its stability and promote glycolytic flux, producing more ATP (Wolf et al., 2011). HKII overexpression is correlated with poor prognosis in CRC and has been proposed as a therapeutic target due to its importance in glycolysis and cellular survival (Hamabe et al., 2014; Ho and Coomber, 2016; Krasnov et al., 2016).

### 2.1 EGFR/Akt in the Warburg Effect

Xanthohumol is a natural compound extracted from hops (*Humulus lupulus*), and it exerts antitumor effects in CRC cells (Jiang et al., 2018). Liu et al. discovered that xanthohumol inhibited glycolysis in CRC cells via EGFR/Akt/HKII axis (Liu et al., 2019). Xanthohumol directly suppressed the phosphorylation of EGFR and EGFR downstream kinases Akt to downregulate the activity of HKII, resulting in a lower rate of glycolysis and activation of mitochondrial-induced apoptosis in CRC cells (Liu et al., 2019). Overexpression of HKII reversed the inhibitory effect of xanthohumol and increased the glycolytic rate in CRC cells (Liu et al., 2019). Epidermal growth factor receptor (EGFR), also known as ErbB1/Her1, is a member of the membrane-bound receptor tyrosine kinase family, which can be activated by binding of its specific ligands, namely epidermal growth factor (EGF) and transforming growth factor α (TGFα) (Wang, 2017). EGFR is overexpressed in many cancers to function as an oncogene (Nishimura et al., 2015; Cairns et al., 2018). Additionally, EGFR can be mutated in cancers to become constitutively active without ligand binding (Cairns et al., 2018). When activated, EGFR phosphorylates the downstream effectors and signaling pathways to initiate a wide range of oncogenic activities such as cell survival and proliferation (Templeton et al., 2014; Cairns et al., 2018; Fernandes et al., 2018).

Meanwhile, Akt is a serine/threonine kinase frequently upregulated in tumor cells, activated by a wide range of growth factors and receptor stimuli, such as EGFR, PI3K, and PTEN (Nitulescu et al., 2018). Akt mainly functions as a central anabolic and survival effector to regulate cellular metabolism and survival through the phosphorylation of target molecules at different cellular compartments (Song et al., 2019; Hoxhaj and Manning, 2020). The EGFR/Akt signaling cascade has been identified as a crucial oncogenic regulator in various cancers, including non-small cell lung cancer(NSCLC) (Chandrasekaran et al., 2017) and prostate cancer (Gan et al., 2010). Additionally, the direct interaction between HKII and the EGFR/Akt pathway could be essential to modulate cancer progression. Another study by Zou et al. reported that the ubiquitin-like protein FAT10 promoted bladder cancer progression by upregulating HKII via the EGFR/Akt pathway (Zou et al., 2021). FAT10 stabilizes EGFR expression by reducing its degradation and ubiquitination (Zou et al., 2021). Similarly, the EGFR/Akt/HKII regulatory circuit modulates the CRC metabolism. Thus, this axis could become a therapeutically significant target for CRC.

### 2.2 C-MYC Signaling in the Warburg Effect

The c-MYC is a member of the MYC family that includes MYCN and MYC (Rahl and Young, 2014). MYC encodes the Myc transcription factor, which dimerizes with Max to bind DNA and regulate about 15 percent of total gene expression in human cells (Dang et al., 2006). The c-MYC expression is tightly regulated by growth factor-dependent signals in normal cells (Dong et al., 2020). However, c-MYC is a proto-oncogene overexpressed in many cancers, including CRC (Gabay et al., 2014). The c-MYC dysregulation in cancers is mainly induced through gene amplification, chromosome translocation, super-enhancer activation, and loss of upstream repressors to stabilize the c-MYC protein expression (Dong et al., 2020; Wolf and Eilers, 2020). c-MYC functions as a critical regulator of malignant transformation by promoting multiple processes, including cell proliferation, cell growth, and genomic instability (Gabay et al., 2014). More importantly, c-MYC contributes to metabolic reprogramming via several effective mechanisms, such as glycolysis, glutaminolysis, mitochondrial biogenesis, and lipid synthesis (Wahlstrom and Henriksson, 2015). Accumulated evidence suggests that c-MYC-driven metabolic reprogramming in cancer cells is mainly characterized by increased uptake of precursors, enhanced rate of glycolysis and glutaminolysis, as well as increased synthesis of fatty acids and nucleotides (Cunningham et al., 2014; Broecker-Preuss et al., 2017; Cai et al., 2019; Casciano et al., 2020). Tang et al. reported that lncRNA GLCC1 regulates CRC progression and glucose metabolism by stabilizing c-MYC to promote the transcription of glycolytic genes in CRC cells (Tang et al., 2019). GLCC1 was significantly upregulated in glucose-depleted CRC cells and bound explicitly with heat shock protein 90 (HSP90) chaperon upon glucose depletion (Tang et al., 2019). GLCC1 modulates the interaction between HSP90 and c-MYC complex to stabilize c-MYC from degradation (Tang et al., 2019). Indirectly, GLCC1 coordinates the localization and binding pattern of c-MYC genome-wide, promoting the expression of LDHA to upregulate glycolytic metabolism for CRC proliferation (Tang et al., 2019). Lactate dehydrogenase A (LDHA) is a cytosolic enzyme encoded by the LDHA gene on the short p arm of chromosome 11 (11p15.4), which plays a critical role in anaerobic and aerobic glycolysis (the Warburg effect) (Kolappan et al., 2015). LDHA facilitates the interconversion of pyruvate to lactate coupled with the recycling of NAD^+^ from NADH during the last step of glycolysis (Blanco and Blanco, 2017b). LDHA is frequently upregulated in CRC and identified as a direct target gene of the c-MYC oncogenic transcription to reprogram cancer metabolism (Wang et al., 2015; Satoh et al., 2017).

Similarly, N-MYC downstream-regulated gene 2 (NDRG2), a well-known tumor suppressor, inhibits glycolysis in CRC cells by modulating c-MYC expression (Xu et al., 2015). NDRG2 is widely expressed in normal tissue and downregulated in various tumors, including CRC (Kloten et al., 2016; Zhang et al., 2017; Morishita et al., 2021). Overexpression of NDRG2 suppresses cellular growth, proliferation, and invasion in cancers (Li et al., 2013; Hong et al., 2016; Yang CL. et al., 2018; Kang et al., 2020). In CRC metabolism, NDRG2 inhibits c-MYC expression by suppressing β-catenin, the critical effector of the Wnt signaling pathway (Xu et al., 2015). Under normal conditions, β-catenin can be degraded via upstream regulators, such as adenomatous polyposis coli (APC), GSK-3β, and casein kinase 1α (CK1α) (Duchartre et al., 2016). When Wnt signaling is often dysregulated in cancers, possibly due to APC mutation, cytoplasmic β-catenin translocates to the nucleus to activate c-MYC transcription (Duchartre et al., 2016). NDRG2 directly inhibits β-catenin to repress the c-MYC expression at the transcriptional level (Xu et al., 2015). c-MYC is the oncogenic transcriptional factor responsible for modulating the Warburg effect in CRC cells through the regulation of glucose transporters (GLUTs) and other glycolytic enzymes, namely HKII, PKM2, and LDHA (Wahlstrom and Henriksson, 2015). NDRG2 suppresses glycolysis in CRC cells by downregulating the expression of GLUT1, HKII, PKM2, and LDHA through c-MYC inhibition (Xu et al., 2015).

Another carcinogenic modulator, CD36, is a membrane glycoprotein that is associated with elevated fatty acids absorption to modulate cancer progression and metastasis in various cancers such as ovarian (Ladanyi et al., 2018), cervical (Yang P. et al., 2018), liver (Nath et al., 2015), and stomach (Pan et al., 2019). However, CD36 has been reported to target β-catenin/c-MYC-mediated glycolysis to repress CRC tumorigenesis (Fang et al., 2019). Early evidence showed that Wnt signaling could play a role in modulating the Warburg effect in CRC cells via the nuclear accumulation of β-catenin (Pate et al., 2014). CD36 was significantly downregulated in CRC tissue, and its expression level was negatively associated with cancer progression (Fang et al., 2019). Ectopic expression of CD36 directly promoted proteasome-dependent ubiquitination of Glypican 4 (GPC4), which is a member of the heparan sulfate proteoglycans (HSPGs) family (Zhao et al., 2016; Fang et al., 2019). Previous studies suggest that lipid raft localization of GPC4 is required to activate the Wnt/β-catenin pathway (Fico et al., 2012; Sakane et al., 2012; Cao et al., 2018). GPC4 degradation reduced the nuclear translocation of β-catenin, leading to the downregulation of c-MYC and downstream glycolytic genes (GLUT1, LDHA, HK2, and PKM2) in CRC cells (Fang et al., 2019). Collectively, CD36 promotes the ubiquitination of GPC4 to de-activate β-catenin/c-MYC signaling cascades and downstream glycolytic target genes, repressing the glycolysis and tumorigenesis in CRC cells (Fang et al., 2019).

HK II is another downstream effector of c-MYC, which could be targeted to modulate glycolysis in CRC cells. Dioscin is a kind of steroid saponins isolated from *Dioscoreae rhizome* and *Paridis rhizome*, which has potent activities against various cancers, including CRC (Si et al., 2016; Li et al., 2018; Mao et al., 2019). A recent study by Wu et al. showed that dioscin inhibits glycolysis and induces apoptosis in CRC cells by targeting c-MYC and HKII (Wu et al., 2020). Upon treatment with dioscin, the interaction between FBW7 and c-MYC in CRC cells was enhanced, leading to the ubiquitination of c-MYC (Wu et al., 2020). Consequently, dioscin promoted c-MYC degradation, and the downstream HKII was suppressed, resulting in glycolysis inhibition (Wu et al., 2020). Dioscin also impaired the interaction between HKII and VDAC-1 on the outer mitochondrial membrane, in which Bax could bind to the VDAC-1 more efficiently (Wu et al., 2020). This phenomenon increased membrane permeability and the release of cytochrome C, which ultimately led to cellular apoptosis (Wu et al., 2020). Thus, HKII is essential for dioscin-mediated glycolysis inhibition and apoptosis in CRC cells.

### 2.3 STAT3 Signaling in the Warburg Effect

Apart from EGFR/Akt and c-MYC signaling pathways, other pathways target HKII to modulate the glucose metabolism in CRC cells. The polo-like kinases (PLKs) belong to a family of highly conserved serine/threonine kinases, in which all five members (PLK1–5) possess a conserved N-terminal kinase domain and one or more polo-box domains (PBDs) at the C-terminus (Barr et al., 2004). Initially, PLKs were found to be critical regulators of cell cycle checkpoint, mitosis, and DNA damage response (Xie et al., 2005). Recent studies suggest that PLKs can modulate tumor growth, apoptosis, and metabolism (Ou et al., 2016; Gutteridge et al., 2017). Ou et al. revealed that PLK3 inhibits glucose metabolism in CRC by targeting HSP90/STAT3/HKII signaling (Ou et al., 2019). PLK3 was significantly downregulated in CRC tissues and correlated with poor prognosis (Ou et al., 2016; Ou et al., 2019). PLK3 was directly bound to HSP90 to trigger proteasome-mediated degradation of HSP90, which reduced the phosphorylation of signal transducer and activator of transcription 3 (STAT3) at S727 residue (Ou et al., 2019). STAT3 directly binds to the promoter region of the HKII gene and upregulates the protein expression HKII. The STAT3 dephosphorylation downregulated the transcriptional activation of HKII, resulting in a lower glycolytic rate in CRC cells (Ou et al., 2019). The STAT family consists of seven proteins (STAT 1, 2, 3, 4, 5a, 5b, and 6), which have dual roles as signaling molecules and transcription factors (Villarino et al., 2015). STATs transduce signals from activated cytokine and growth factor receptors into the nucleus to initiate the transcription of target genes (Villarino et al., 2015). Specific cytokines and growth factors activate each STAT protein to regulate downstream target genes. For instance, cytokine receptors activate receptor-associated tyrosine kinases, such as the Janus kinase (JAK) family kinases, to phosphorylate the tyrosine 705 residue (Y705) in STAT3 protein (Parganas et al., 1998). Growth factor receptors associated with their intrinsic receptor tyrosine kinase (RTK) phosphorylate STAT3 at the same tyrosine 705 residue (Y705).

STAT3 is activated in response to a defined set of cytokines, namely IL-6, interferon-gamma (IFNγ), and erythropoietin, in addition to growth factors including epidermal growth factor (EGF) and fibroblast growth factor (FGF) (O'Sullivan et al., 2016). Tyrosine phosphorylation activates STAT3 to undergo homo/hetero-dimerization with another STAT protein(O'Sullivan et al., 2016). STAT3 dimers bind to specific DNA response elements in the promoter regions of target genes to regulate the gene transcription (O'Sullivan et al., 2016). Activated STAT3 dimer induces the expression of multiple genes associated with anti-apoptosis, proliferation, angiogenic, and metastatic properties in cancer cells (Timofeeva et al., 2013; Liu et al., 2015; Zhu et al., 2019). Moreover, serine/threonine kinases phosphorylate STAT3 on S727 in the cytoplasm or nucleus (Zhang et al., 1995). The serine phosphorylation of STAT3 is required for maximal transcriptional activity but not DNA binding (Wen et al., 1995). Multiple kinases are responsible for S727 phosphorylation, more prominently the mitogen-activated protein kinase (MAPK) family members (Shaheen and Broxmeyer, 2018). The MAPK family consists of the extracellular signal-regulated kinase (ERK) as well as JNK/stress-activated protein kinase and p38/HOG1 (p38 MAPK) (Shaheen and Broxmeyer, 2018). STAT3 signaling is well studied as a significant intrinsic pathway for cancer inflammation due to its frequent activation in cancer cells that promotes inflammatory genes and suppresses anti-tumor immunity (Yu et al., 2009). Nevertheless, recent studies suggest that STAT3 signaling contributes to metabolic reprogramming in cancers. Lin et al. reported that palmitic acid inhibits glycolysis in hepatocellular carcinoma by silencing the STAT3 pathway (Lin et al., 2017). STAT3 signaling is also essential in PKM2-mediated glucose metabolism in breast cancer cells via the let-7a-5p/Stat3/hnRNP-A1 regulatory feedback loop (Yao A. et al., 2019).

It was observed that STAT3 signaling plays a crucial role in the PLK3-inhibited glucose metabolism of CRC cells by targeting HKII expression (Ou et al., 2019). The interplay between STAT3 signaling and c-MYC has altered glycolysis in CRC cells in response to inflammation (Qu et al., 2017). IL-6 is a pro-inflammatory cytokine frequently detected in the tumor microenvironment to induce inflammation and activate STAT3 signaling in cancer cells (Yu et al., 2009; Kim et al., 2016; Arora et al., 2018). *In vitro* studies revealed that the addition of IL-6 into CRC cell lines activated the phosphorylation of STAT3 and increased the expression of c-MYC and glycolytic enzymes, such as GLUT1 and LDH, resulting in higher glucose uptake and lactate production (Qu et al., 2017). These results suggest that inflammation could induce the reprogramming of glucose metabolism in CRC cells via the STAT3/c-MYC pathway. JAK2 is an upstream regulator of STAT3, which phosphorylates STAT3 at the Y705 residue. A recent study by Li et al. identified that JAK2/STAT3 signaling was targeted by atractylenolide-I to induce apoptosis and suppress glycolysis in CRC cells (Li Y. et al., 2020). Atractylenolide-I (AT-I) is a natural derivative of *Rhizoma Atractylodis* macrocephalus that has been shown to demonstrate anti-tumor activities in a wide range of cancers. Mechanistically, AT-I could directly bind to JAK2 to inhibit the JAK2 activity and suppress the downstream phosphorylation of STAT3 (Li Y. et al., 2020). Subsequently, the inactivation of STAT3 contributed to the downregulation of HKII, resulting in a lower rate of glycolysis and lactate production in CRC cells (Li Y. et al., 2020). Hence, AT-I inhibits glycolysis via JAK2/STAT3 signaling to suppress HKII expression in CRC cells (Li Y. et al., 2020).

### 2.4 The PKM2 Paradox in the Warburg Effect

Pyruvate kinase (PK) is a rate-limiting enzyme in the final, irreversible step of the glycolysis, which is responsible for catalyzing the transphosphorylation between phosphoenolpyruvate and ADP to generate pyruvate and ATP (Blanco and Blanco, 2017b). There are four mammalian PK isoforms: PKL, PKR, PKM1, and PKM2, each with distinct kinetic properties and tissue distribution (Clower et al., 2010). PKL is mainly expressed in the liver and kidneys, while PKR is exclusively expressed in red blood cells (Israelsen et al., 2013). PKM1 is primarily expressed in differentiated tissues with high energetic demands, such as myocardium, skeletal muscle, and brain tissue (Chiavarina et al., 2011). PKM2 is distributed in tissues, such as the brain and liver, and is highly expressed in rapidly proliferating tissues, including cancers (Shiroki et al., 2017). The PK isoforms are encoded by two genes (PKLR and PKM), respectively, through the alternative splicing of pyruvate kinase mRNA (PKL and PKR; PKM1 and PKM2) (Chen et al., 2010). The human PKM gene with a length of 12 exons is alternatively spliced to generate transcripts based on the mutually exclusive selection between 9th and 10th exons: exon 9 is specific to PKM1, exon 10 is specific to PKM2 (Israelsen and Vander Heiden, 2015). Multiple splicing factors regulate the PKM1/PKM2 ratio in cancerous tissue, in which PKM2 is more favorable in most cancer types to modulate the Warburg effect (Israelsen et al., 2013). For instance, polypyrimidine tract binding protein (PTB), heterogeneous nuclear ribonucleoprotein A1 (HNRNPA1), and A2 (HNRNPA2B1) repress exon 9 and promote exon 10 to upregulate the PKM2 expression (Clower et al., 2010). Another splicing factor, namely the serine/arginine-rich splicing factor 3 (SRSF3), directly binds the PKM transcript to promote the inclusion of exon 10 for enhancing the PKM2 expression (Chen et al., 2010). Previous evidence also suggests that c-MYC activates the expression of HNRNPs to maintain a high PKM2/PKM1 ratio in cancer cells (David et al., 2010).

PKM2 exists in two oligomeric states: an active tetramer and a less active dimer/monomer, due to tetramerization upon binding with fructose-1,6-bisphosphate (FBP) (Sciacovelli et al., 2014). The moderately active, dimer form of PKM2 mainly participates in the Warburg effect in cancers by producing glycolytic intermediates to support tumor growth and proliferation. The PKM2 dimers also induce transcriptional co-activation and function as protein kinase targeting histones and transcription factors (Lu, 2012). PKM2 dimers translocate into the cell nucleus upon signaling from the extracellular signal-regulated kinase (ERK1/2) to initiate the expression of other glycolytic genes (GLUT1, LDHA, PDK) (Yang et al., 2012). The nuclear translocation of PKM2 is crucial for the autoregulation of PKM2 expression by upregulating its upstream activators, such as HIF1α and β-catenin (Luo et al., 2011; Yang et al., 2012; Prigione et al., 2014). More importantly, nuclear PKM2 interacts with HIF1α and β-catenin to regulate the expression of glycolytic enzymes and initiate the Warburg effect in cancer cells (Luo et al., 2011; Yang et al., 2011). In contrast, the tetramer form of PKM2 is fully active to maximize the efficiency of glycolysis and generate pyruvate for the utilization of oxidative phosphorylation but is excluded from nuclear translocation (Prakasam and Bamezai, 2018).

Recently, Sam68, an RNA-binding protein (RBP), has been identified to regulate glycolysis in CRC cells by controlling the alternative splicing of the PKM gene (Zhao J. et al., 2020). Sam68 is well recognized as a critical oncogenic factor associated with cancer progression and poor prognosis in CRC (Liao et al., 2013; Fu et al., 2016; Wang et al., 2018). Ectopic expression of Sam68 upregulates the glycolysis and proliferation in CRC cells, associated with decreased PKM1/PKM2 ratio (Zhao J. et al., 2020). Sam68 binds to the EI9 region of the PKM gene to promote the inclusion of exon 9 and enhance the formation of PKM2 mRNA (Zhao J. et al., 2020). Overexpression of Sam68 significantly reduces the PKM1/PKM2 ratio in CRC cells, resulting in the metabolic shift from oxidative phosphorylation to glycolysis (Zhao J. et al., 2020). Sam68 increases the PKM2 mRNA transport into the cytoplasm to enhance the PKM2 protein synthesis, promoting the pyruvate kinase activity and lactate production in CRC cells (Zhao J. et al., 2020). In addition, polypyrimidine tract binding protein 1 (PTB1) is a positive regulator of the Warburg effect in cancer cells by regulating the PKM2 expression (Clower et al., 2010). PTB1 is an exonic splicing silencer of the PKM mRNA that promotes the PKM2 expression by including exon 10 in alternative splicing (He et al., 2014). c-MYC can regulate PTB1 to promote cancer progression and the Warburg effect (David et al., 2010). Taniguchi et al. have presented several works on the regulatory role of miR-124 on PTB1 and PKM1/PKM2 ratio in modulating the Warburg effect in CRC (Taniguchi et al., 2015a; Taniguchi et al., 2015b). MiR-124 was downregulated in CRC clinical samples, while *in vitro* analysis revealed that miR-124 induced apoptosis and suppressed the Warburg effect in CRC cells (Taniguchi et al., 2015b). PTB1 promotes the production of PKM2, which inhibits miR-124 in a feedback loop (Taniguchi et al., 2015b). MiR-124 induces the switching of PKM isoforms from PKM2 to PKM1 by downregulating PTB1 and its upstream regulators, namely c-MYC, E2F1, and STAT3 (Taniguchi et al., 2015b).

In addition, miR-124 can also regulate the Warburg effect in CRC cells via the DDX6/c-MYC/PTB1 positive-feedback mechanism (Taniguchi et al., 2015a). DDX6 is an oncogenic RNA helicase frequently overexpressed in multiple cancers, including CRC (Akao et al., 2006; Cordin et al., 2006; Akao, 2009; Tajirika et al., 2018). DDX6 expression is associated with the IRES-dependent c-MYC translation to regulate cancer cell growth and differentiation (Hashimoto et al., 2001; Akao et al., 2006; Taniguchi et al., 2018). DDX6 is significantly overexpressed in CRC tissues, in which the authors suggest that low expression of miR-124 contributes to the high DDX6 expression (Taniguchi et al., 2015a). MiR-124 directly targets DDX6 in CRC cells, in which miR-124 knockdown releases DDX6 to promote the c-MYC expression, and c-MYC upregulates PTB1 directly, contributing to the Warburg effect (Taniguchi et al., 2015a). PTB1 knockdown upregulates the miR-124 expression, subsequently suppressing the expression of DDX6 and c-MYC and inhibiting the Warburg effect in CRC cells (Taniguchi et al., 2015a). All this evidence supports that PTB1 is an essential modulator of CRC metabolism that can be regulated by miRNAs and oncogenic upstream activators, like c-MYC, to modulate the expression of PKM1 and PKM2 in the Warburg effect. Evidence shows that modulation of the Warburg effect involving the metabolic role of PKM2 could lead to the acquisition of other cancer phenotypes, such as chemoresistance. CD44 is a non-kinase transmembrane glycoprotein frequently overexpressed in cancers and cancer stem cells (Chen et al., 2018). CD44 functions as an adhesion molecule in many aspects of tumorigenesis, including migration, proliferation, and metastasis, as well as to function as a surface marker for cancer stem cells (Du et al., 2008; Liu et al., 2011; Louderbough and Schroeder, 2011; Cho et al., 2012; Wang et al., 2012; Senbanjo and Chellaiah, 2017). Overexpression of CD44 directly phosphorylates PKM2 at threonine (T105) residue to suppress its glycolytic activity and promote the Warburg effect in CRC cells (Tamada et al., 2012). CD44 knockdown also induces the metabolic shift from aerobic glycolysis to mitochondrial respiration with increased reactive oxygen species (ROS) production, which significantly re-sensitizes CRC cells towards cisplatin (Tamada et al., 2012). Hence, PKM2 is crucial for the CD44-mediated Warburg effect with enhanced cisplatin resistance in CRC cells.

Multiple signaling pathways regulate the metabolic role of PKM2 in CRC cells. The Wnt/β-catenin signaling pathway is aberrantly activated in CRC due to the adenomatous polyposis coli (APC) gene mutation (loss of function) in nearly 90 percent of CRC patients (Coppede et al., 2014). APC protein is required to form the β-catenin destruction complex for β-catenin degradation and inhibition of the Wnt/β-catenin signaling pathway (MacDonald et al., 2009). The loss of function in the APC gene is commonly associated with the early transformation of normal colon epithelium into adenoma (Powell et al., 1992). Mutant APC protein cannot form the destruction complex, leading to stabilization and accumulation of β-catenin expression in the cytosol (Requena and Garcia-Buitrago, 2020). The subsequent nuclear translocation of β-catenin activates inappropriate target genes, namely c-MYC and cyclin D, associated with tumor proliferation, migration, invasion, and metastasis (Nguyen and Duong, 2018). More recently, the APC mutation in CRC cells has been reported to induce the Warburg effect via the Wnt/β-catenin signaling pathway to target PKM2 (Cha et al., 2021). The loss of function in APC stabilizes the β-catenin and increases the nuclear translocation of β-catenin (Cha et al., 2021). Subsequently, this enhances the β-catenin/Tcf4 binding on PKM2 promoter regions and promotes PKM2 transcription (Cha et al., 2021). The PKM2 upregulation further increases the expression of other glycolytic enzymes, namely LDHA, GLUT1, PFK1-M, and PFKBP1, to enhance the Warburg effect in CRC cells (Cha et al., 2021). PKM2 also accelerates the activation of Wnt/β-catenin signaling via a positive feedback loop in CRC cells (Cha et al., 2021). Collectively, the Wnt/β-catenin signaling activated by APC mutation requires PKM2 as the critical mediator to modulate the Warburg effect in CRC.

**FIGURE 1:**
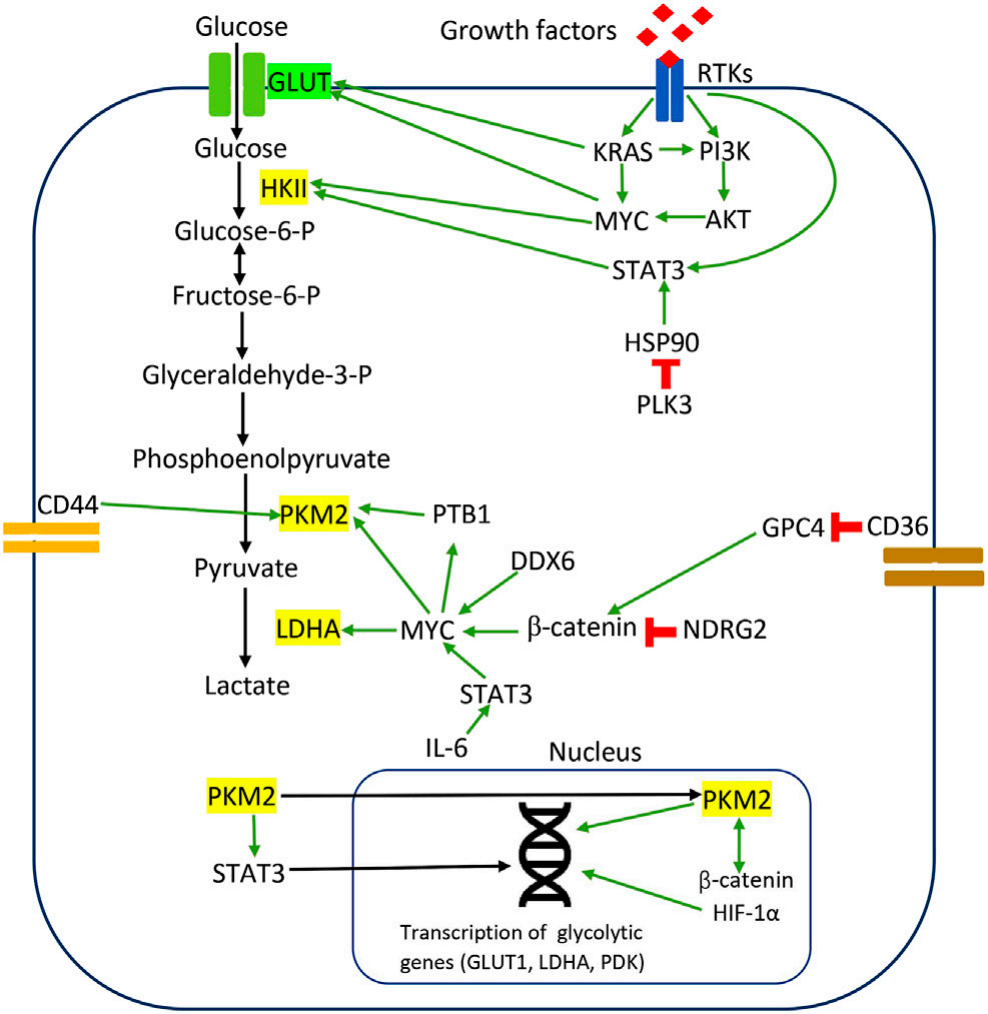
Regulation of the Warburg effect via oncogenic signaling in CRC. The red sign indicates inhibition, while the green arrow indicates promotion.

In CRC, PKM2 induces direct phosphorylation of transcription activators and signaling molecules to mediate metabolic reprogramming and proliferation. Previous studies observed that PKM2 phosphorylated STAT3 at Tyr705 to activate STAT3-controlled genes, namely MEK5, for cell proliferation (Gao et al., 2012). Yang et al. reported that PKM2 facilitates CRC cell migration via the STAT3 signaling cascade (Yang P. et al., 2014). PKM2 overexpression in DLD1 cells upregulated STAT3 gene transcription and activated downstream snail-2 and β1-integrin-FAK signaling to induce tumor migration (Yang P. et al., 2014). PKM2 overexpression facilitated STAT3 nuclear translocation to upregulate the expression and function of PKM2 in migration and adhesion-associated signaling, suggesting the feedback mechanism between PKM2 and STAT3 (Yang P. et al., 2014). These results demonstrate that the protein kinase activities of dimeric PKM2 but not its metabolic functions are essential for CRC cell migration and cell adhesion. Figure 1 illustrates the regulation of the Warburg effect via oncogenic signaling in CRC.

### 3 PKM2 in Glutaminolysis

Glutamine is the most abundant non-essential amino acid in most cancer cells, which is highly required for cellular proliferation (Sciacovelli et al., 2014). Glutamine is catalyzed by glutaminase (GLS) to generate glutamate and converted into other intermediates, namely α-ketoglutarate, pyruvate, lactate, and malate, which can be utilized in different metabolic pathways (Blanco and Blanco, 2017a). Hence, glutamine metabolism or glutaminolysis is equally essential in the metabolic reprogramming of CRC cells by supporting ATP production and biosynthesis of proteins, lipids, and nucleic acids. The less glycolytic, dimer form of PKM2 is crucial to coordinate the metabolism change between glycolysis and glutaminolysis in CRC cells (Li L. et al., 2020). PKM2 dimer facilitates glutaminolysis upon EGFR stimulation by promoting IRES-dependent c-MYC translation (Li L. et al., 2020). Previously, c-MYC regulates PKM2 expression by controlling PKM2 pre-mRNA splicing, and subsequently, PKM2 regulates c-MYC gene transcription in a direct feedback mechanism (David et al., 2010; Luo et al., 2011; Yang et al., 2012). The recent work by Li et al. demonstrates that PKM2 interacts with the c-MYC/IRES complex to regulate c-MYC translation via another IRES-dependent mechanism (Li L. et al., 2020). There are two pathways in c-MYC protein synthesis at the translational level: canonical cap-dependent translation and internal ribosome entry site (IRES)-dependent translation (Godet et al., 2019). The IRES-dependent c-MYC translation is an oncogenic pathway that allows the c-MYC protein synthesis under critical conditions, such as apoptosis and hypoxia, when the canonical cap-dependent translation is largely attenuated (Stoneley et al., 2000; Spriggs et al., 2009). As PKM2 dimer promotes IRES-dependent c-MYC translation in CRC cells, the activated c-MYC protein subsequently upregulates GLS-I to increase glutamine consumption (Li L. et al., 2020). Glutaminase I (GLS-I) is the mitochondrial enzyme that catalyzes the first, irreversible step of glutaminolysis by converting glutamine to glutamate (Jin et al., 2016). GLS-I plays a vital role in glutaminolysis and is frequently dysregulated in cancers (Pan et al., 2015; Daemen et al., 2018; Xiang et al., 2019; Ren et al., 2020). The less active form of PKM2 dimer modulates glutaminolysis in CRC cells by targeting IRES-dependent c-MYC translation to coordinate cell response to hypoxia environment. The oligomeric switching between tetramer and dimer forms of PKM2 corresponds to the metabolic change between glycolysis and glutaminolysis in CRC cells upon the stimulation of growth factors and environmental stress.

Another study proposed that PKM2 depletion could promote the β-catenin signaling and its downstream c-MYC to enhance glutamine metabolism in CRC cells (Wu et al., 2014). CRC cells enhance glutamine metabolism to compensate for glycolysis impairment upon PKM depletion (Wu et al., 2014). Previously, it was shown that c-MYC regulates mitochondrial glutaminase expression and glutamine metabolism in cancer cells (Gao et al., 2009). Interestingly, PKM2-knockdown in CRC cells significantly upregulates c-MYC protein expression and β-catenin expression at mRNA and protein levels, suggesting that PKM2 regulates glutaminolysis mainly via β-catenin/c-MYC signaling (Wu et al., 2014). Nuclear translocation of dimeric PKM2 negatively regulates the β-catenin mRNA at the transcriptional level through the action of miR-200a that directly targets the 3’ UTR of β -catenin mRNA (Wu et al., 2014). Collectively, PKM2 functions as the protein kinase that negatively affects the β-catenin/c-MYC signaling pathway through miR-200a to modulate glutaminolysis in CRC cells.

### 4 KRAS Mutations and PI3K Signaling in Glutamine Metabolism

KRAS mutation is common in many cancers, including CRC (Ewing et al., 2014). KRAS mutation causes the protein to become constitutively active AND promote the signaling through growth and survival pathways, namely the phosphatidylinositol 3-kinase (PI3K) and mitogen-activated protein kinase (MAPK) cascades (Kerk et al., 2021). Mutant KRAS has been used as a clinical biomarker to predict resistance to anti-epidermal growth factor receptor (EGFR) therapy in treating metastatic CRC (Kudryavtseva et al., 2016). Previous studies suggest that KRAS mutation could be essential to modulate glucose uptake, glutaminolysis, and mitochondrial ROS production in CRC metabolism. Yun et al. reported that CRC cell lines with mutations in either KRAS or BRAF upregulate GLUT-1 with increased glucose uptake and lactate production (Yun et al., 2009). Another proteomic study showed that mutant KRAS and BRAF in CRC cell lines could impact multiple aspects of metabolism, including glycolysis, phosphoserine biosynthesis, glutamine metabolism, and the non-oxidative pentose phosphate pathway, by modulating the expression of corresponding proteins/enzymes (Hutton et al., 2016). KRAS and BRAF mutations contribute to metabolic reprogramming in CRC to support rapid proliferation and sustain biosynthetic needs. Additionally, it was demonstrated that glutamine-based mitochondrial metabolism is essential for KRAS mutant CRC cells to support cell growth and proliferation (Weinberg et al., 2010). KRAS mutant CRC cells acquire a high glycolytic flux to provide glycolytic intermediates for the pentose phosphate pathway to produce nucleotides and phospholipids for rapid proliferation (Weinberg et al., 2010). Glutamine metabolism provides the alternative carbon source for the TCA cycle in mitochondrial respiration to generate ATP for cellular proliferation and tumorigenesis (Weinberg et al., 2010).

Knockdown of the glutamine transporter in KRAS mutant CRC cells reduces the proliferation rate and inhibits other oncogenic activities, including migration, invasion, and metastasis (Wong et al., 2016). A recent study by Wong et al. also reveals that glutamine metabolism in mutant KRAS CRC cells contributes to the activation of Wnt signaling, cancer stemness, and drug resistance by reducing DNA methylation through SLC25A22, a mitochondrial glutamine transporter (Wong et al., 2020). SLC25A22 expression is associated with poor prognosis in advanced-stage CRC with mutant KRAS (Wong et al., 2020). SLC25A22 is upregulated in mutant KRAS CRC cells to induce succinate accumulation in the cell nucleus and subsequently modulate epigenetic regulators' expression to enhance WNT/β-catenin signaling and LGR5 expression (Wong et al., 2020). Indirectly, SLC25A22 promotes cancer stemness and drug resistance in CRC cells.

Phosphoinositide 3-kinases (PI3Ks) comprise a large family of lipid kinases that function as intracellular signal transducers (Noorolyai et al., 2019). PI3K signaling can be activated through the upstream RAS isoforms, tyrosine kinase receptors, and mutations in PI3K signaling components (43). The activation of the PI3K pathway is mainly associated with cell cycle progression, proliferation, differentiation, and metabolism in cancer cells (Fernandes et al., 2018). PIK3CA gene encodes the p110α catalytic subunit of PI3K, and its mutation represents one of the most common genetic aberrations in human cancers (Zardavas et al., 2014). PI3KCA mutations reprogram glutamine metabolism in CRC cells by upregulating glutamate pyruvate transaminase 2 (GPT2) (Hao et al., 2016). Consequently, CRC cells with PI3KCA mutations are more sensitive to glutamine deprivation and substantially increase glutamine metabolism to replenish the TCA cycle and generate ATP (Hao et al., 2016). Mutant p110α upregulates GPT2 gene expression through novel PDK1-RSK2-ATF4 signaling that is AKT-independent (Hao et al., 2016). Mutant p110α activates RSK2 kinase through pyruvate dehydrogenase kinase 1 (PDK1) (Hao et al., 2016). PDK1 is a downstream effector of PI3K via the signal transduction from phosphatidylinositol-3,4,5-triphosphate (PIP3), whereas RSK2 is a serine/threonine kinase that phosphorylates ATF4 at the serine 245 residue (S245) (Frodin et al., 2002; Hao et al., 2014). Activated RSK2 phosphorylates ATF4 to recruit the deubiquitinase USP8, preventing ATF4 from ubiquitin-mediated degradation (Hao et al., 2016). ATF4 (activating transcription factor 4) has been reported to modulate glutamine metabolism in different cancer studies (Qing et al., 2012; Csibi et al., 2013). Eventually, ATF4 activates GPT2 gene transcription directly to promote PI3K-mediated glutamine metabolism (Hao et al., 2016).

## 5 Signaling Pathways in Oxidative Phosphorylation

KRAS mutation is also associated with the regulation of mitochondrial respiration in CRC cells. Mitochondrial respiration comprises two major components: the tricarboxylic acid cycle (TCA cycle) and oxidative phosphorylation (OXPHOS) (Blanco and Blanco, 2017c). TCA cycle occurs in the inner mitochondrial space to generate NADH and FADH2 via acetyl-CoA metabolism (Blanco and Blanco, 2017b). The NADH and FADH_2_ generated by the TCA cycle will be oxidized in OXPHOS, which takes place on the inner membrane of mitochondria, to generate ATP and reactive oxygen species (ROS) as a byproduct (Blanco and Blanco, 2017b). In CRC, KRAS mutations have decreased ROS production and enhanced mitochondrial OXPHOS efficiency by activating mitochondrial phospholipid synthesis via the upregulation of transcriptional factors HIF-1α and HIF-2α (Chun et al., 2010). The hypoxia-inducible factors-1α and -2α (HIF-1α and HIF-2α) are frequently upregulated in cancers and associated with tumor angiogenesis, cell growth and survival, and metastasis (Li T. et al., 2020). It was shown that HIF-1α and HIF-2α proteins translocate to the nucleus and dimerize with HIF-1β to transactivate target genes (Koh et al., 2011). Previously, HIF-1α and HIF-2α regulated the exchange of COX4 (cytochrome c oxidase 4) subunits under hypoxic conditions to increase mitochondrial respiration efficiency and reduce ROS production (Fukuda et al., 2007).

## 6 Signaling Pathways in Lipid Metabolism

Dysregulation of lipid metabolism is equally essential for CRC tumor growth and survival. Fatty acids are essential components of all biological membranes and the critical carbon source for energy metabolism in the TCA cycle and OXPHOS (Blanco and Blanco, 2017d). Fatty acids can act as signaling molecules associated with multiple aspects of tumorigenesis, such as migration, invasion, and drug resistance (Nath et al., 2015; Blanco and Blanco, 2017c; Ladanyi et al., 2018; Pan et al., 2019). In CRC, multiple studies have shown that the *de novo* synthesis of fatty acids is enhanced with the upregulation of critical enzymes, including ATP citrate lyase (ACLY), acetyl CoA carboxylase (ACC), and fatty acid synthase (FASN) (Zaytseva et al., 2012; Hofmanova et al., 2021). Upregulation of FASN has been reported in mutant KRAS cell lines, which supports cellular respiration through mitochondrial fatty acid β-oxidation (FAO) (Zaytseva et al., 2015). FAO is the primary pathway for degrading long-chain fatty acids to produce acetyl-CoA, which participates in the TCA cycle to replenish ATP, NADPH, NADH, and FADH_2_ (Houten et al., 2016). A recent study by Wang et al. demonstrates that epigallocatechin-3-gallate (EGCG), a water-soluble polyphenol and the main active ingredient of green tea, inhibits fatty acid *de novo* synthesis and lipid droplets formation in CRC cells via AMPK activation (Wang et al., 2021). AMP-activated protein kinase (AMPK) is a serine/threonine protein kinase, which serves as a metabolic sensor and core regulator of energy metabolism in cancers (Steinberg and Carling, 2019). Previously, AMPK activation could decrease *de novo* lipogenesis (Smith and Steinberg, 2017). In addition, studies have shown that AMPK activation inhibits the Warburg effect and *de novo* lipogenesis while increasing OXPHOS to suppress cancer cell growth (Chen et al., 2012; Smith and Steinberg, 2017; Holczer et al., 2018). EGCG significantly increases the phosphorylation of AMPKα (Thr172) to inhibit the expression of FASN, ACLY, ACC, and the transcriptional factor sterol regulating element-binding protein 1c (SREBP1c), resulting in *de novo* synthesis inhibition and reduced cell viability in CRC cells (Wang et al., 2021).

## 7 Signaling Pathways in Tryptophan Metabolism

**FIGURE 2:**
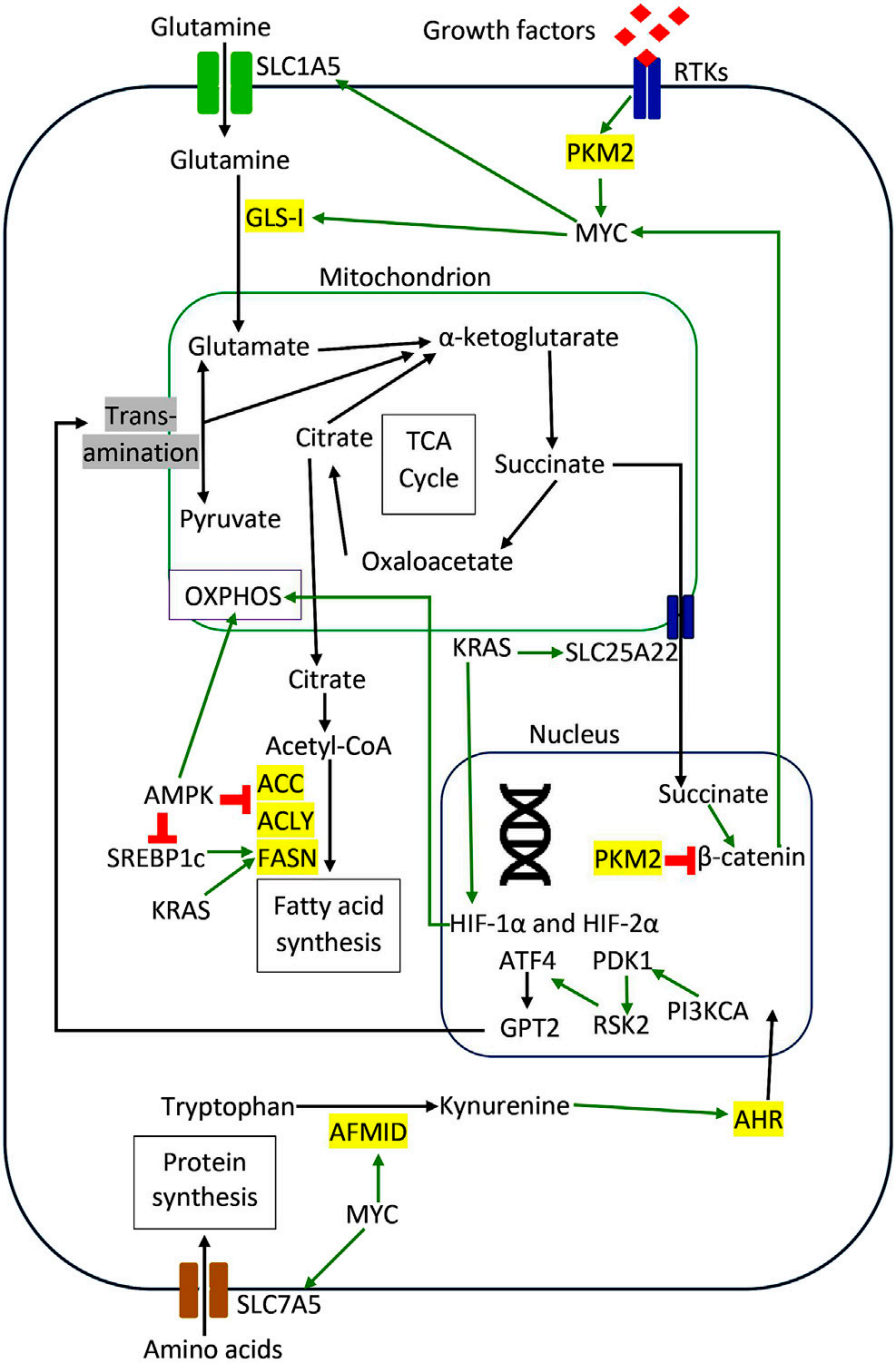
Regulation of amino acids and lipid metabolism via oncogenic signaling in CRC cells. The red sign indicates inhibition, while the green arrow indicates promotion.

There are many other types of amino acid metabolism in cancer cells. Tryptophan (Trp) is an essential amino acid for protein synthesis, and it is the least abundant amino acid in most proteins (Adams et al., 2012). Venkateswaran et al. have presented a comprehensive work on the tryptophan metabolism in CRC cells. They discovered that CRC cells increase the tryptophan uptake and metabolism compared to normal colonic cells and tissues (Venkateswaran et al., 2019). Similarly, CRC cells were more sensitive to tryptophan depletion than their normal counterpart (Venkateswaran et al., 2019). Tryptophan can be metabolized in three different pathways (Blanco and Blanco, 2017a). Firstly it is incorporated into newly synthesized proteins (Blanco and Blanco, 2017a). Secondly, it enters the serotonin pathway to produce serotonin and melatonin (Blanco and Blanco, 2017a). Lastly, tryptophan enters the kynurenine pathway to generate kynurenine (Kyn), a biologically active metabolite (Van der Leek et al., 2017). The proto-oncogene c-MYC activates the kynurenine pathway in CRC cells by promoting the transcription of the tryptophan importers SLC1A5 and SLC7A5 and the tryptophan metabolizing enzyme arylformamidase AFMID (Venkateswaran et al., 2019). In the kynurenine pathway, tryptophan is metabolized by one of the three enzymes: indoleamine 2,3-dioxygenase1 (IDO1), indoleamine 2,3-dioxygenase2 (IDO2), and tryptophan 2,3-dioxygenase 2 (TDO2) to generate N-formyl kynurenine, which is converted into kynurenine by AFMID (Van der Leek et al., 2017). Previously, IDO1 expression correlates with impaired immune response, hepatic metastases, and poor clinical outcomes in CRC (Brandacher et al., 2006). IDO1 and kynurenine pathway metabolites activate PI3K-AKT signaling to promote nuclear translocation of β-catenin, enhancing cancer cell proliferation and inhibit apoptosis in CRC cells. (Thaker et al., 2013; Bishnupuri et al., 2019). SLC7A5, SLC1A5, and AFMID were upregulated in CRC clinical samples with the increased level of kynurenine (Venkateswaran et al., 2019). Kynurenine functions as an oncometabolite to activate the nuclear translocation of the transcription factor AHR (aryl hydrocarbon receptor), which regulates target genes associated with proliferation in CRC cells (Venkateswaran and Conacci-Sorrell, 2020). Figure 2 illustrates the regulation of amino acids and lipid metabolism via oncogenic signaling in CRC cells. Table 1 summarizes the main contributions of previous studies to the field of CRC metabolism, as discussed earlier.

**TABLE 1:** Summarizes the main contributions of previous studies to the field of CRC metabolism.

Molecule/Signaling pathway involved	Target enzyme/Effector	Function	Reference
The Warburg Effect			
EGFR/Akt	HKII	Inhibits glycolysis upon suppression by xanthohumol	Liu et al. (2019)
GLCC1/c-MYC	LDHA	Promotes CRC progression and glucose metabolism	Tang et al. (2019)
NDRG2/β-catenin/c-MYC	GLUT1, HKII, PKM2, and LDHA	Inhibits glycolysis through c-MYC suppression	Xu et al. (2015)
CD36/GPC4/β-catenin/c-MYC	Multiple glycolytic genes	Inhibits glycolysis and tumorigenesis in CRC cells	Fang et al. (2019)
CD44	PKM2	Promotes Warburg effect	Tamada et al. (2012)
Nuclear PKM2/HIF1α/β-catenin	Multiple glycolytic genes	Promotes glycolysis and autoregulation of PKM2	Luo et al. (2011), Yang et al. (2012), Prigione et al. (2014)
IL-6/STAT3/c-MYC	GLUT1, LDH	Increases glucose uptake and lactate production	Qu et al. (2017)
JAK2/STAT3	HKII	Inhibits glycolysis and lactate production	Li et al. (2020c)
c-MYC	HKII	Inhibits glycolysis and induce apoptosis in CRC cells upon dioscin stimulation	Wu et al. (2020)
PLK3/HSP90/STAT3	HKII	Inhibits glucose metabolism	Ou et al. (2019)
Sam68	PKM1/PKM2	Upregulates glycolysis and proliferation in CRC cells	Zhao et al. (2020a)
miR-124/c-MYC/E2F1/STAT3/PTB1	PKM1/PKM2	Promotes PKM2 production and glycolysis	Taniguchi et al. (2015b)
miR-124/DDX6/c-MYC/PTB1	PKM2	Promotes Warburg effect	Taniguchi et al. (2015a)
Wnt/β-catenin/PKM2	LDHA, GLUT1, PFK1-M, and PFKBP1	Promotes Warburg effect	Cha et al. (2021)
Glutaminolysis			
EGFR/PKM2/c-MYC/IRES	GLS-I	Promotes glutaminolysis	Li et al. (2020a)
KRAS mutation	Multiple glutaminolysis enzymes	Regulates glutaminolysis, mitochondrial respiration, and ROS production	Weinberg et al. (2010), Hutton et al. (2016)
KRAS mutation	Mitochondrial glutamine transporter SLC25A22	Regulates glutamine metabolism, Wnt signaling, cancer stemness, and drug resistance	Wong et al. (2016)
Nuclear PKM2/miR-200/β-catenin/C-MYC	Mitochondrial glutaminase	Promotes glutaminolysis	Wu et al. (2014).
PI3K/PDK1/RSK2/ATF4	GPT2	Increases glutamine metabolism to replenish the TCA cycle and generate ATP	Hao et al. (2016)
Oxidative Phosphorylation (OXPHOS)			
KRAS mutations	HIF-1α and HIF-2α	Increases mitochondrial respiration and reduces ROS production	Chun et al. (2010)
Lipid Metabolism			
AMPK	FASN, ACLY, ACC, SREBP1c	Inhibits fatty acid synthesis and reduces cell viability	Wang et al. (2021).
KRAS mutations	FASN	Upregulates fatty acid β-oxidation	Zaytseva et al. (2015)
Tryptophan Metabolism			
C-MYC	SLC7A5, SLC1A5, AFMID	Upregulates tryptophan metabolism via the kynurenine pathway	Venkateswaran et al. (2019)

## 8 Metabolic Reprogramming in Colorectal Cancer Cancer Stem Cells

Cancer stem cells (CSCs) represent a subpopulation of tumor cells with self-renewal and multi-lineage differentiation to regulate tumor growth and heterogeneity (Walcher et al., 2020). CSCs have been identified as the critical regulator of cancer progression in various solid tumors, including CRC, driving cancer initiation and cancer relapse (Ayob and Ramasamy, 2018). CSCs undergo metabolic adaptation to support their stemness properties and promote tumor development (Batlle and Clevers, 2017). In CRC, the metabolic reprogramming in CSCs is closely associated with the expression of signaling molecules and stem cell markers. CRC CSCs are known to express specific stem cell markers, such as CD133, CD44, leucine-rich repeat-containing G protein-coupled receptor 5 (LGR5), and epithelial cell adhesion molecule (EpCAM) (Munro et al., 2018). CD133 is a well-characterized marker in CRC CSCs, while CD133(+) cells can initiate tumor formation in the animal model (Wang et al., 2012; Li, 2013). Based on the analysis of microarray data from the GEO database, which included sorted CD133(+) and CD133(−) subfractions of CRC CSCs, Chen et al. revealed that genes involved in glycolysis, TCA cycle, and one-carbon metabolism were upregulated in CD133(+) cells. However, at the same time, genes involved in fatty acid biosynthesis were downregulated (Chen et al., 2014). Their findings suggest that CD133(+) CRC CSCs upregulates glycolysis for energy production while suppressing fatty acid biosynthesis.

CD133(+) CRC CSCs also exhibit an altered lipid metabolism as compared to non-CSC counterparts. By using transmission electron microscopy imaging and flow cytometry analysis, Tirinato et al. discovered that CD133(+) CSCs contain more lipid droplets in the cytoplasm as compared to CD133(−) cells (Tirinato et al., 2015). The authors also observed a similar result by stratifying cells based on Wnt/β-catenin activity instead of CD133 expression level, suggesting a possible correlation between CD133 and Wnt signaling to modulate the lipid metabolism in CRC stem cells (Tirinato et al., 2015). Functionally, CRC stem cells with high lipid droplet content demonstrated a higher *in vitro* sphere-forming ability and a more significant tumor formation upon subcutaneous injection in the animal model. The reprogramming of lipid metabolism could be essential in promoting the tumorigenesis of CRC stem cells.

CD44 is another stem cell marker in CRC CSCs, which also acts as the cell surface glycoprotein to participate in cellular interactions and cellular migration (Morath et al., 2016; Senbanjo and Chellaiah, 2017). CD44 transcription is activated by β-catenin/Wnt signaling, while its overexpression is frequently associated with the early transformation of colorectal adenoma to carcinoma (Orian-Rousseau, 2015). Knockdown of CD44 in CRC cell line HCT116 decreased the glucose uptake and consumption by downregulating PKM2 activity and lactate production (Tamada et al., 2012). Simultaneously, CD44 ablation in HCT116 cells also reduced glucose utilization by the pentose phosphate pathway (Tamada et al., 2012). CD44 could play an essential role in the metabolic reprogramming of CRC stem cells by inducing the switching of glucose metabolism from OXPHOS to glycolysis and pentose phosphate pathways. Moreover, CD44(+) CRC CSCs express a higher glutamine level as compared with the CD44(−) counterparts, but the actual interaction between CD44 and glutamine metabolism is yet to be investigated (Huang et al., 2016). The increased glutamine in CD44(+) CSCs can support CRC energy production to promote cell survival, growth, and proliferation.

The transcription factor p63 is a member of the p53 transcription factor family, which has been expressed in CRC CSCs, while p63 mutation is associated with poor clinical prognosis among CRC patients (Pignon et al., 2013; Bahnassy et al., 2014). Through its N-terminal *trans*-activator domain, p63 activates mitochondrial glutaminase 2 (GLS2) in CRC cells to catalyze the conversion of glutamine into glutamate and α-ketoglutarate for subsequent ATP production via the TCA cycle (Giacobbe et al., 2013). Glutamate is an essential precursor of glutathione, which is the primary ROS intracellular scavenger. The authors observed that GLS2 was significantly upregulated in CRC tissue samples with decreased cellular levels of ROS, suggesting that p63/GLS2 axis could be essential to protect CRC cells against oxidative stresses (Giacobbe et al., 2013). Furthermore, p63 overexpression in CRC is also associated with the upregulation of glycolysis in CSC cultures, implicating that p63 could be a crucial glycolytic regulator in CRC stem cells (D’Aguanno et al., 2014).

## 9 Crosstalk Between Oncogenic Signaling and Metabolic Pathways in Colorectal Cancer Progression

CRC pathogenesis is a multi-step transformation that involves multiple genetic alterations to promote cancer initiation and progression. The activation of oncogenic signaling is essential to drive the stepwise progression of CRC via metabolic reprogramming to promote cancer cell survival, proliferation, epithelial-mesenchymal transition (EMT), angiogenesis, invasion, and migration. Metabolic reprogramming plays a vital role in different stages of CRC progression, from the early transformation of normal epithelial cells into the entire adenoma-carcinoma sequence. Lgr5^+^ intestinal stem cells (ISCs) are the progenitors of secretory cells and enterocytes that produce mature intestinal epithelial cells, so ISCs are also the cancer stem cells for CRC, as shown by lineage tracing studies (Barker et al., 2009; Merlos-Suarez et al., 2011; Kemper et al., 2012). TIGAR (TP53-inducible glycolysis and apoptosis regulator) is transcriptionally activated by p53 tumor suppressor protein to decrease the intracellular levels of fructose-2,6-bisphosphate (Fru-2,6-BP) and downregulate the glycolytic pathway (Bensaad et al., 2006). Subsequently, TIGAR diverts the glucose metabolism towards the pentose phosphate pathway (PPP) to produce NADPH for antioxidant function and ribose-5-phosphate for nucleotide synthesis (Li and Jogl, 2009). Eric et al. reported that TIGAR was required for injury-induced regeneration in ISCs, by promoting PPP to generate NADPH and ribose-5-phosphate rapidly (Cheung et al., 2013). TIGAR was upregulated in human CRC cell lines and tumors regardless of p53 status, while the authors also observed that TIGAR promoted adenoma formation in a mouse model of intestinal cancer (Cheung et al., 2013). All this evidence suggests that TIGAR is essential to support cell proliferation by reprogramming glucose metabolism during CRC initiation.

Additionally, reprogramming of lipid metabolism is also associated with CRC initiation and maintenance of ISCs. Prolonged high-fat diet (HFD) in mice activates PPAR-δ (peroxisome proliferator-activated receptor-delta) in ISCs to upregulate a subset of β-catenin target genes associated with cancer cell stemness (Beyaz et al., 2016). Consequently, ISCs proliferate rapidly and increase adenoma formation in a mouse model of APC loss-induced intestinal tumorigenesis (Beyaz et al., 2016). Kim et al. reported that loss of PKM2 in Lgr5(+) ISCs promoted inflammation-associated CRC in the mouse model, while a similar result was also reported in the APC-driven colon cancer mouse model (Lau et al., 2017; Kim et al., 2019). These studies suggest that PKM2 expression is not required for CRC initiation, although PKM2 overexpression was reported in advanced stages of CRC (Lau et al., 2017). The downregulation of PKM2 also significantly led to the activation of PKM1, which may suggest a compensatory expression of PKM1 by deletion of PKM2 to support the metabolic requirement and proliferation of CRC cells (Kim et al., 2019). Meanwhile, Satoh et al. describe that c-MYC is responsible for inducing a global metabolic reprogramming in CRC, which starts from the adenoma stage and remains similar through all cancer stages (Satoh et al., 2017). c-MYC induces the upregulation of glycolysis and nucleotide metabolism to support the rapid proliferation of CRC cells during tumorigenesis (Satoh et al., 2017). Additionally, the protein kinase function of PKM2 activates c-MYC upon the signaling from RTKs (Li L. et al., 2020). The activated c-MYC also increases the glutamine uptake and metabolism in CRC cells by promoting the glutamine transporter SLC1A5 and glutaminase GLS-I (Li L. et al., 2020).

**FIGURE 3:**
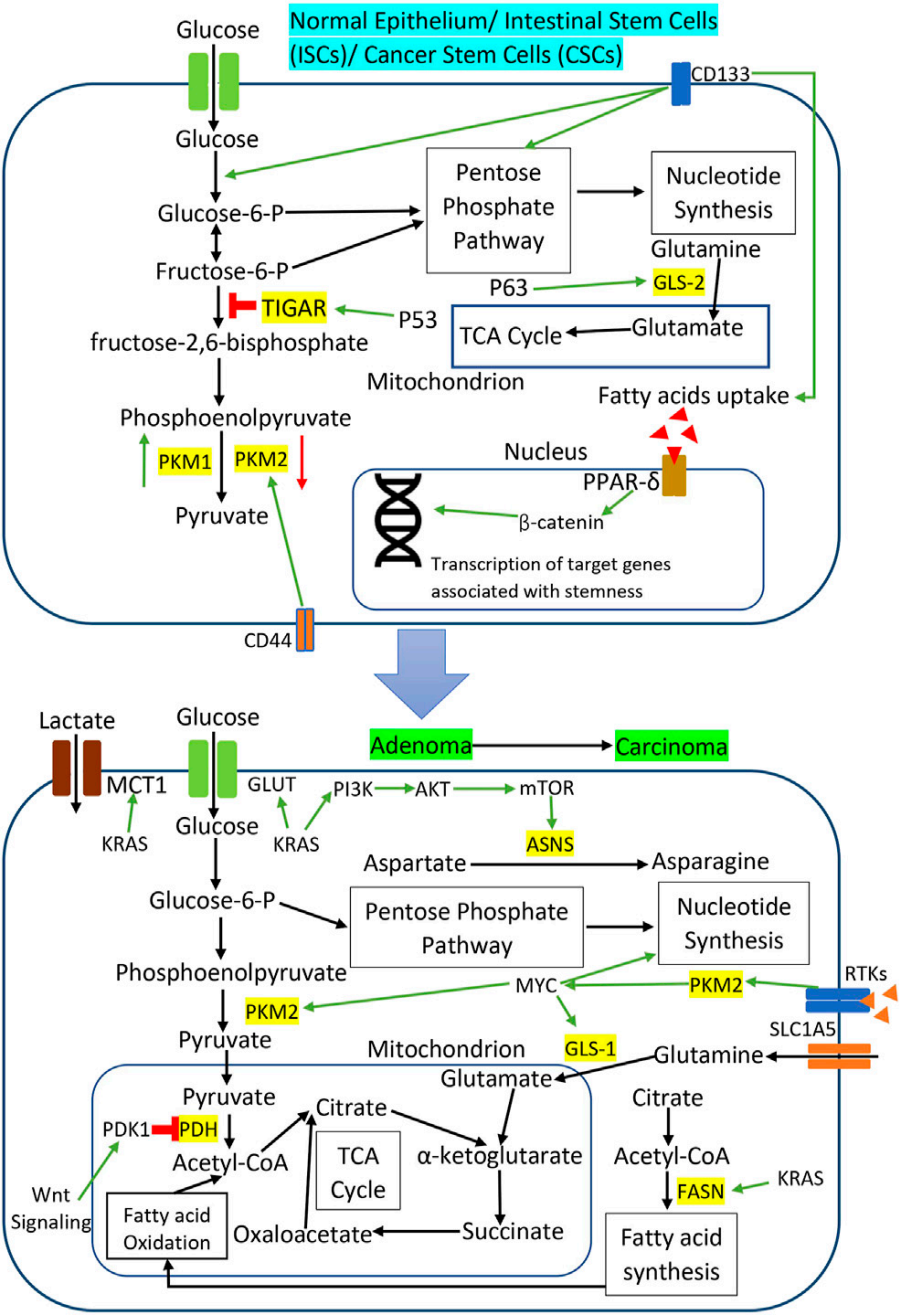
Metabolic reprogramming through oncogenic signaling during CRC initiation and progression as well as metabolic reprogramming in CRC stem cells. The red sign indicates inhibition while the green arrow indicates promotion.

Accumulating evidence suggests that Wnt signaling and KRAS mutations induce metabolic reprogramming in the adenoma and carcinoma stage of CRC to support the energetic and biosynthetic requirements of rapidly proliferating CRC cells. Activation of Wnt signaling in CRC cell lines promotes glucose metabolism and lactate production by upregulating the transcription of pyruvate dehydrogenase kinase 1 (PDK1) and lactate transporter, MCT-1 (Pate et al., 2014; Sprowl-Tanio et al., 2016). Wnt signaling-induced PDK1 prevents pyruvate flux to mitochondrial respiration and indirectly promotes cancer proliferation and angiogenesis in xenograft CRC tumors in the mouse model (Pate et al., 2014). KRAS mutations in CRC also promote tumor growth and cancer progression by rewiring glucose, amino acids, and lipid metabolism. KRAS mutations increase the glucose uptake in CRC cells via the upregulation of GLUT1 to generate more glycolytic intermediates for the utilization in different metabolic pathways in tumor expansion (Kawada et al., 2012; Iwamoto et al., 2014). Toda et al. observed that KRAS mutation altered the amino acid metabolism in CRC cells by reducing aspartate level and increasing asparagine level to maintain cell viability and tumor growth under the glutamine-depleted condition (Toda et al., 2016). Mutant KRAS upregulated asparagine synthetase (ASNS) expression through PI3K/AKT/mTOR signaling to increase the asparagine biosynthesis and promote CRC cell growth (Toda et al., 2016). Meanwhile, fatty acid synthase (FASN) is overexpressed in KRAS mutant CRC cell lines to support cellular respiration via lipid oxidation and subsequently provide a survival advantage under metabolic stress (Zaytseva et al., 2015). Figure 3 illustrates specific metabolic pathways rewired through oncogenic signaling during CRC initiation and progression, as well as metabolic reprogramming in CRC stem cells.

## 10 Targeting Colorectal Cancer Metabolism

As discussed above, metabolic reprogramming is critical in the CRC tumorigenesis to support cancer initiation, proliferation, invasion, and metastasis. The first line of treatment for early-stage CRC patients (stage I and II) is surgical resection, while chemotherapy and radiotherapy are mostly recommended for late-stage II and above (Dekker et al., 2019). Chemotherapy for CRC patients usually includes the combination of chemotherapy agents, such as FOLFOX (5-FU, leucovorin, and oxaliplatin) or FOLFIRI (5-FU, leucovorin, and irinotecan), to maximize the treatment efficacy (Keum and Giovannucci, 2019). However, chemotherapy often comes with serious side effects, such as nausea, vomiting, loss of hair, and body weakness, which affect the life quality of CRC patients (Onyoh et al., 2019). Drug resistance becomes another difficulty in chemotherapy for CRC, as it quickly gives rise to cancer relapse and metastasis that significantly reduce the survival rate and prognosis among CRC patients (Rawla et al., 2019). Over the years, researchers have been looking into alternatives for chemotherapy in CRC management, possibly with more minor side effects and similar efficacy. Thus, targeting CRC metabolism could become another potential option for CRC therapy.

The advancement in the molecular biology of cancer has allowed researchers to identify the dysregulation of protein kinases as the effectors of signaling pathways in cancer development and progression. These relevant findings have led to pharmacological inhibitors that directly target protein kinases and signaling pathways in cancers to kill the cancer cells more effectively (Butti et al., 2018; Jiang and Ji, 2019). As protein kinases are the essential regulators of metabolic reprogramming, protein kinases inhibitors could be repurposed to target the CRC metabolism. In general, most of the inhibitors available can be grouped into two categories: small molecule intervention and antibody blocking (Lemmon and Schlessinger, 2010). Small-molecule inhibitors can target the ATP-binding site of protein kinases specifically to inhibit phosphorylation, while monoclonal antibodies are developed to bind to the extracellular domain of protein kinases and suppress kinase-ligand interaction (Gaumann et al., 2016; Gan et al., 2017). Many of these inhibitors are being investigated in phase II clinical studies in CRC, while the US FDA has approved several drugs for targeted therapy in clinical application, as listed in Table 2. These small molecule inhibitors and monoclonal antibodies can be used as monotherapy or combined with conventional therapies to increase the therapeutic efficacy and cancer specificity (To et al., 2020; Hwang et al., 2021). For instance, cetuximab and panitumumab are monoclonal antibodies that target EGFR and can be used as monotherapy or combined with chemotherapy to treat patients with RAS wild-type metastatic CRC (Hayashi et al., 2017; Garcia-Foncillas et al., 2019).

**TABLE 2:** List of small molecule inhibitors and monoclonal antibodies for CRC therapy in clinical trials or approved by the FDA.

Drug	Target	Clinical stage	Reference
*Small Molecule Inhibitors*			
Afatinib	EGFR	Phase II	De Pauw et al. (2016)
Alpelisib	PI3K	Phase II	Tabernero et al. (2016)
Cobimetinib	MAPK	Phase II	Klute et al. (2020)
Dabrafenib	BRAF	Phase II	Corcoran et al. (2015)
Dasatinib	Src	Phase II	Scott et al. (2017)
Encorafenib	BRAF	FDA approval in 2020	Kopetz et al. (2019)
Enzastaurin	AKT	Phase II	Wolff et al. (2012)
Erlotinib	EGFR	Phase III	Hagman et al. (2016)
Everolimus	mTOR	Phase II	Ng et al. (2013)
Gedatolisib	PI3K/mTOR	Phase II	Wainberg et al. (2017)
Gefitinib	EGFR	Phase II	Troiani et al. (2016)
Mk-2206	AKT	Phase II	Do et al. (2015)
Napabucasin	STAT3	Phase III	Jonker et al. (2018)
Niclosamide	STAT3	Phase I	Burock et al. (2018)
Pelitinib	EGFR	Phase II	To et al. (2020)
Regorafenib	VEGFR, KIT, PDGFR, RET, TIE2,EPH2A	FDA approval in 2012	Papadopoulos and Lennartsson, (2018)
Sonolisib	PI3K	Phase II	Bowles et al. (2016)
Trametinib	BRAF	Phase II	Corcoran et al. (2015)
Temsirolimus	mTOR	Phase II	Spindler et al. (2013)
Vemurafenib	BRAF	Phase II	Klute et al. (2020)
*Monoclonal Antibodies*			
Bevacizumab	VEGF-A	FDA approval in 2004	Rosen et al. (2017)
Cetuximab	EGFR	FDA approval in 2004	Bokemeyer et al. (2012)
Panitumumab	EGFR	FDA approval in 2006	Hocking and Price, (2014)
Pertuzumab	HER2	Phase II	Meric-Bernstam et al. (2019)
Ramucirumab	VEGFR2	FDA approval in 2015	Verdaguer et al. (2016)
Rilotumumab	HGF	Phase II	Van Cutsem et al. (2014)
Trastuzumab	HER2	Phase II	Meric-Bernstam et al. (2019)

Some natural compounds and synthetic agents have been discovered to target metabolic enzymes and signaling molecules directly. Consequently, these compounds can function as metabolic inhibitors which could potentially target CRC metabolism, as listed in Table 3. Some studies demonstrate that naturally derived metabolic inhibitors can be administered without side effects and are much safer than conventional chemotherapeutics (Pandurangan and Esa, 2014; Wang et al., 2021). Obesity and a high-fat diet are commonly associated with a higher risk of CRC, in which upregulation of lipid metabolic enzymes such as FASN and ACC often leads to CRC progression and metastasis through activation of oncogenic pathways including Wnt, PI3K/AKT, AMPK/mTOR. Thus, research on compound targeting enzymes involved in lipid metabolism could be promising in the future management of CRC cases. For example, luteolin (3,4,5,7-tetrahydroxyflavone) which is found in vegetables, fruits, and medicinal herbs, functions as a fatty acid synthase (FASN) inhibitor to modulate lipid metabolism in CRC cells via Wnt/β-catenin signaling (Pandurangan and Esa, 2014). Luteolin can be orally administrated in a dosage of up to 500 mg twice a day without side effects (Luo et al., 2017). Notably, the combination of metabolic inhibitors and chemotherapy drugs has been shown to enhance the therapeutic efficacy and reduce the toxicity simultaneously, which is promising for the future development of CRC therapy. For instance, a combination of glutaminase (GLS) inhibitor CB839 and 5-FU chemotherapy drug increases the anti-cancer effect on the xenograft growth of PIK3CA-mutant CRC cells without significant dose-limiting toxicity (Zhao Y. et al., 2020).

**TABLE 3:** List of potential metabolic inhibitors that target CRC cells.

Compound	Target molecule/pathway	Function	Reference
2-DG	Glycolytic enzymes	Inhibition of glycolysis with reduction of tumor invasion and metastasis	Park et al. (2017), Xiang et al. (2019)
DON	Multiple glutamine-utilizing enzymes	Inhibition of glutaminolysis and induction of cellular ROS	Lemberg et al. (2018); Schcolnik-Cabrera et al. (2020)
AZD3965	MCT1	Inhibition of lactate transport, glycolysis, and lipid biosynthesis	Beloueche-Babari et al. (2020)
CB839	GLS1	Inhibition of glutaminolysis	Zhao et al. (2020b), Cohen et al. (2020)
Cerulenin plus oxaliplatin	FASN	Inhibition of proliferation and metastasis with induction of apoptosis	Murata et al. (2010); Shiragami et al. (2013)
EGCG	FASN	Downregulation of STAT3 and reduced proliferation	Wang et al. (2021)
GSK165	ACLY	Activation of AKT signaling	Zhou et al. (2013)
IDF-11774	HIF1α	Inhibition of glycolysis and angiogenesis	Misale et al. (2012)
L-Aspartate plus Rapac-MYCin	ASNS and mTOR	Inhibition of asparagine biosynthesis	Toda et al. (2016)
Luteolin	FASN	Modulation of Wnt/β-catenin signaling	Horinaka et al. (2005), Pandurangan and Esa (2014), Yao et al. (2019b)
Metformin	Mitochondrial electron transport complex I	AMPK activation and PKM2 inhibition	Zhang et al. (2011); Carr et al. (2016)
Mito-Metformin	Mitochondrial electron transport complex I	ATP depletion	Hosono et al. (2010)
Oxamate	LDHA	Inhibition of glycolysis	Yang et al. (2014b), Salgado-Garcia et al. (2021)
RO5126766	RAF/MEK	Downregulation of GLUT1	Wu et al. (2015)
TVB-3166	FASN	Inhibition of *de novo* palmitate synthesis	Ventura et al. (2015)
TVB-3664	FASN and CD36	Suppression of lipid metabolism and transport, Modulation of AKT and ERK1/2 signaling	Zaytseva et al. (2018)
Vitamin C	EGFR/MAPK	Decreased phosphorylation of PKM2 and reduced GLUT1 expression	Aguilera et al. (2016)
WZB117	GLUT1	Downregulation of GLUT1	Misale et al. (2012)
WZB117 plus Ficlatuzumab	GLUT1 and HGF	Inhibition of glucose uptake and blocking HGF activity	Song et al. (2017)

## 11 Conclusion

The reprogramming of cellular metabolism is vital in tumor development. Metabolic reprogramming constitutes an important cancer hallmark in CRC, which gives rise to the alteration in aerobic glycolysis, glutaminolysis, OXPHOS, and lipid metabolism. Numerous studies have demonstrated that oncogenic mutations and loss of tumor suppressor genes contribute to CRC cell metabolic reprogramming by modulating the downstream signaling pathways. Protein kinases, such as AKT and c-MYC, regulate the expression of metabolic enzymes at both transcriptional and translational levels. The crosstalk between oncogenic pathways and the metabolic pathways represents another new avenue to develop therapeutics for CRC. Small molecule inhibitors and monoclonal antibodies currently available could be repurposed to target CRC metabolism by regulating protein kinases and signaling pathways involved. The discovery of metabolic inhibitors could become the alternative to the current treatment options, such as chemotherapy and radiotherapy, that are associated with severe side effects. The development of therapeutic based on CRC metabolism is challenging when most of the research data is generated from cell line models. Given the complexity of the tumor microenvironment and the heterogeneity of actual tumors, many questions regarding dosage safety and the therapeutic efficacy in clinical trials are waiting to be answered.
